# The Impact of Macro-and Micronutrients on Predicting Outcomes of Critically Ill Patients Requiring Continuous Renal Replacement Therapy

**DOI:** 10.1371/journal.pone.0156634

**Published:** 2016-06-28

**Authors:** Kittrawee Kritmetapak, Sadudee Peerapornratana, Nattachai Srisawat, Nicha Somlaw, Narisorn Lakananurak, Thasinas Dissayabutra, Chayanat Phonork, Asada Leelahavanichkul, Khajohn Tiranathanagul, Paweena Susantithapong, Passisd Loaveeravat, Nattachai Suwachittanont, Thaksa-on Wirotwan, Kearkiat Praditpornsilpa, Kriang Tungsanga, Somchai Eiam-Ong, Piyawan Kittiskulnam

**Affiliations:** 1 Division of Nephrology, Department of Medicine, Konkaen University, Konkaen, Thailand; 2 Division of Nephrology, Department of Medicine, Chulalongkorn University, King Chulalongkorn Memorial Hospital, Bangkok, Thailand; 3 Division of Clinical Nutrition, Department of Medicine, Chulalongkorn University, King Chulalongkorn Memorial Hospital, Bangkok, Thailand; 4 Department of Biochemistry, Faculty of Medicine, Chulalongkorn University, King Chulalongkorn Memorial Hospital, Bangkok, Thailand; 5 Department of Microbiology, Faculty of Medicine, Chulalongkorn University, King Chulalongkorn Memorial Hospital, Bangkok, Thailand; Bambino Gesù Children's Hospital, ITALY

## Abstract

Critically ill patients with acute kidney injury (AKI) who receive renal replacement therapy (RRT) have very high mortality rate. During RRT, there are markedly loss of macro- and micronutrients which may cause malnutrition and result in impaired renal recovery and patient survival. We aimed to examine the predictive role of macro- and micronutrients on survival and renal outcomes in critically ill patients undergoing continuous RRT (CRRT). This prospective observational study enrolled critically ill patients requiring CRRT at Intensive Care Unit of King Chulalongkorn Memorial Hospital from November 2012 until November 2013. The serum, urine, and effluent fluid were serially collected on the first three days to calculate protein metabolism including dietary protein intake (DPI), nitrogen balance, and normalized protein catabolic rate (nPCR). Serum zinc, selenium, and copper were measured for micronutrients analysis on the first three days of CRRT. Survivor was defined as being alive on day 28 after initiation of CRRT.Dialysis status on day 28 was also determined. Of the 70 critically ill patients requiring CRRT, 27 patients (37.5%) survived on day 28. The DPI and serum albumin of survivors were significantly higher than non-survivors (0.8± 0.2 vs 0.5 ±0.3g/kg/day, p = 0.001, and 3.2±0.5 vs 2.9±0.5 g/dL, p = 0.03, respectively) while other markers were comparable. The DPI alone predicted patient survival with area under the curve (AUC) of 0.69. A combined clinical model predicted survival with AUC of 0.78. When adjusted for differences in albumin level, clinical severity score (APACHEII and SOFA score), and serum creatinine at initiation of CRRT, DPI still independently predicted survival (odds ratio 4.62, p = 0.009). The serum levels of micronutrients in both groups were comparable and unaltered following CRRT. Regarding renal outcome, patients in the dialysis independent group had higher serum albumin levels than the dialysis dependent group, p = 0.01. In conclusion, in critically ill patients requiring CRRT, DPI is a good predictor of patient survival while serum albumin is a good prognosticator of renal outcome.

## Introduction

Acute kidney injury (AKI) is a common complication in critically ill patients. The overall incidence of AKI in intensive care unit (ICU) patients ranges from 25 to 40 percent and contains high mortality rate [1–3].Patients with AKI are generally in high catabolic state due to massive releases of pro-inflammatory cytokines, catabolic hormones, uremic toxin, metabolic acidosis, and insulin resistance [4,5].

Moreover, in severe AKI patients who require continuous renal replacement therapy (CRRT) support, there is a chance to loss a significant amount of macronutrients, especially protein, due to the high permeability property of dialyzer membrane [6]. Provision of adequate dietary protein intake (DPI) may compensate for this high catabolic state and make nitrogen balance become more positive [7,8].Besides macronutrients, micronutrients including trace elements may also play an essential role in various physiologic functions, such as normal protein synthesis, DNA repair, anti-inflammatory functions, and immune regulation in severe AKI patients [9].

However, the outcome association between macro/micronutrients and severe AKI patients undergoing CRRT is still unclear. Studies in the 1990s and early 2000s had used old-fashioned CRRT technique and did not mention the association between nutritional parameters and clinical outcomes [10–12]. In the *post hoc* analysis of Randomized Evaluation of Normal versus Augmented Level (RENAL) study [13], low DPI did not show the association of low DPI and the poor clinical outcome. Therefore, there are still conflicting results in clinical studies.

In this study, we aimed to examine the role of macronutrient and micronutrient parameters in predicting outcomes of AKI patients requiring CRRT.

## Materials and Methods

### Study Population and Setting

The study protocol was approved by The Institutional Review Board of Faculty of Medicine, Chulalongkorn University. All participants accepted the protocol and provided written informed consent.

This was a prospective single-center, cohort study of AKI patients treated by CRRT during November 2012 and November 2013 in the medical and surgical ICU.Eligible participants were adults aged 18 years or older who were admitted in ICU with AKI and required CRRT support. The indications for initiation of CRRT included hemodynamically unstable patients with refractory fluid overload, severe hyperkalemia, severe metabolic acidosis, severe azotemia, and uremic symptoms. Patients with chronic kidney disease (serum creatinine >2 mg/dL in male and >1.5 mg/dL in female), pregnancy, receiving kidney transplant, and those on CRRT for less than 24 hours were excluded. We defined the patients as the survivors if they were still alive on day 28 after initiation of CRRT. “Dialysis independence” was defined as the conditions which the patients still survived and were dialysis independent on day 28 after CRRT initiation. All patients underwent continuous venovenous hemofiltration (CVVH) with the dose of 25 mL/kg/h. The blood flow rate was set at 150–200 mL/min. We used the Polyethersulfone dialyzers (Aquamax HF19^**®**^) during CRRT without using any anticoagulants.

### Clinical Data

In the first 24 hours after CRRT was initiated, the variables including demographic data, severity of illness evaluated according to the Acute Physiology and Chronic Health Evaluation II(APACHE II) score, Sequential Organ Failure Assessment (SOFA) score, primary treating services (medical or surgical), AKI etiologies (ischemic, nephrotoxic, septic or multifactorial), indication of dialysis, renal function at baseline and when initiating dialysis, comorbidity conditions, use of vasopressors, need for mechanical ventilation, vascular access site, nutritional status assessed by subjective global assessment (SGA), nutritional route (enteral or parenteral), and daily dietary DPI, were recorded.

### Blood, Urine, and Effluent Fluid Samples

The patients were evaluated while were in relatively steady metabolic control, usually at least 24 hours after CRRT initiation. The blood samples were serially collected in three consecutive days for measurement of serum albumin, urea, and CRP level. Urine and effluent fluid samples were also daily collected for measurement of urea nitrogen during the first three days of CRRT.Urea nitrogen appearance (UnA) was assessed by multiplying effluent solute concentrations(s) by effluent volume for a given interval of analysis. Urea nitrogen in urine during the period of analysis were also added. UnA was obtained by correcting for weight fluctuations and changes in serum solute concentrations during the same period.

The blood was collected in serum-separating tubes and the serum was separated within 60 minutes of sample collection, then aliquoted and stored at -20 degree Celsius. Serum albumin and urea were measured using the automatic biochemistry analyzer. Serum CRP was measured by nephelometry immunoassay. The blood samples were also assessed for zinc, selenium, and copper for three consecutive days of CRRT by inductively coupled plasma optical emission spectroscopy (Optimal 2100, lPerkin Elmer, USA).

Urea nitrogen was also determined every 12 hours in both effluent and urine (if patients had urine output of >400 mL/day). The effluent volume was measured hourly until CRRT discontinuation[12].

$$\text{UnA}\;=\;\frac{UnMRe\;+\;UnMRu}{60\;\times \;T}\;-\;\frac{BW_{1}\;\times \;\left(Cun_{1}\;-\;Cun_{2}\right)}{10\;\times \;T}\;-\;\frac{Cun_{2}\;\times \;\left(BW_{1}\;-\;BW_{2}\right)}{60\;\times \;T}$$

Where UnA is in milligrams per minute, urea nitrogen eliminated in effluent (UnMRe) and urea nitrogen eliminated in urine (UnMRu) are in milligrams for the entire period, and Cun_1_, Cun_2_, BW_1_, BW_2_, and T indicate respectively serum urea nitrogen concentrations at the beginning of the study period and at the end of the period (both in milligrams per deciliter), body weight at the beginning of the period and at the end of the period (both in kilograms), and finally, the time interval of analysis (in hours). From the UnA parameter, the normalized protein catabolic rate (nPCR in grams per kilograms per 24 hours) was derived according to the equation from Garred et al[12].

$$\text{nPCR}\;=\;\frac{9.35\times UnA}{BW}\;+\;0.17$$

### Statistical Analysis

We compared the baseline characteristics between the survivors and non-survivors. Continuous data were expressed as mean ± standard deviation (SD) and compared using the student’s t-test. Then we demonstrated the level of macro- and micronutrients on the first three days of CRRT.To find out the potential nutritional markers for predicting the outcome, we first compared the mean levels of macro- and micronutrient parameters between the survivors and non-survivors by using the student’s t-test. Finally, we fitted logistic regression model to evaluate the association between the macronutrient parameters and the non-survivor and generated a receiver operating characteristic curve (ROC) and calculated the area under the ROC (AUC-ROC) to assess model discrimination.

## Results

**Fig 1:**
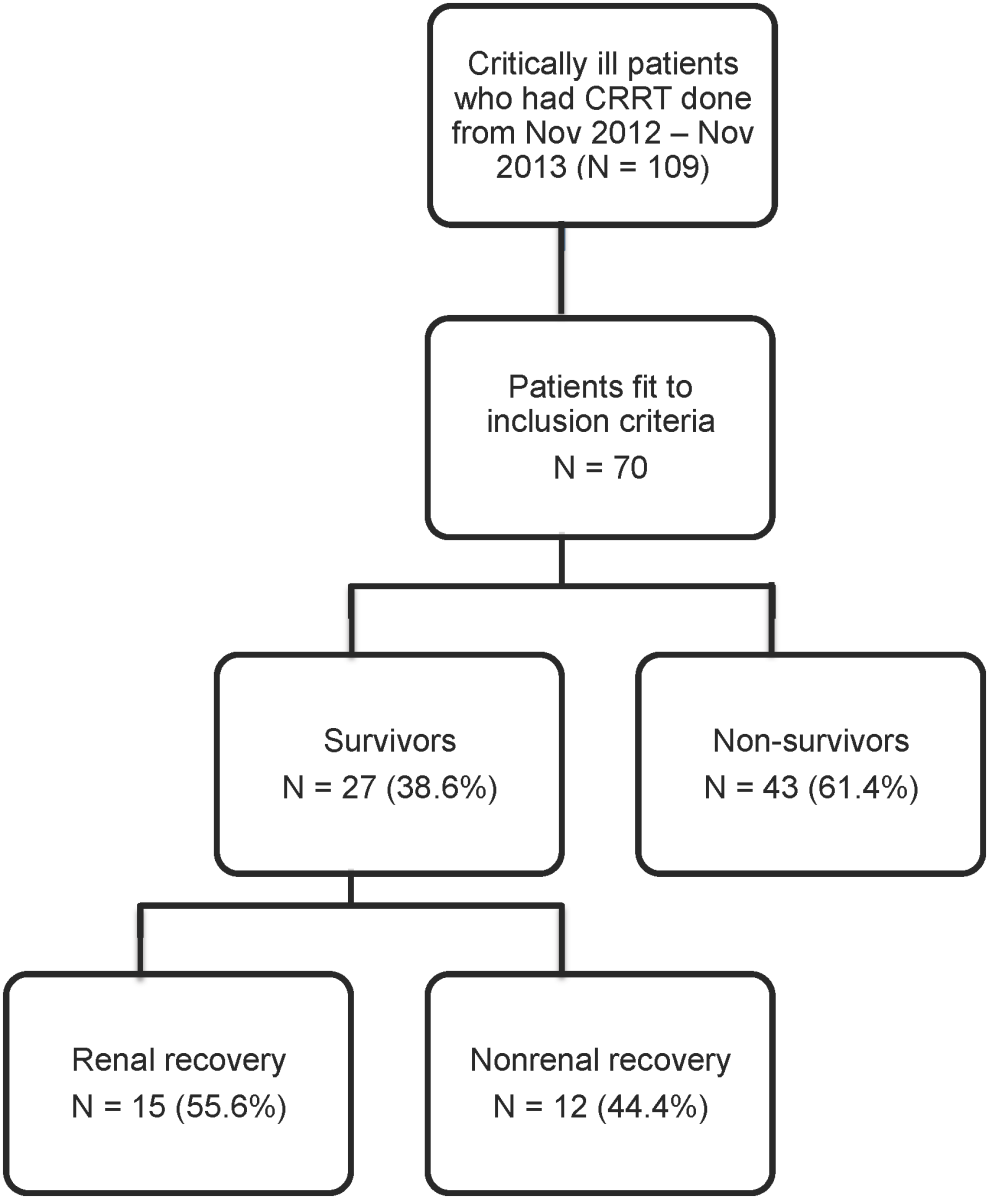
Subject disposition for the study cohort.

Seventy patients with AKI requiring CRRT were included into the study. Twenty seven (38.6%) survived on day-28. For the survivor group, 15 patients had renal recovery (Fig 1). The baseline characteristics between survivors and non-survivors were shown in Table 1. The mean values of body mass index (BMI) were comparable between two groups. The survivor group had significant higher serum creatinine at the time of CRRT initiation than the non-survivor group, 4.07±1.61 vs 3.26±1.19 mg/dL, p = 0.01. Ischemic process was the leading cause of AKI in the survivors while sepsis was the leading cause of AKI in the non-survivors. Oliguria was the most common indication to start CRRT in both groups. The survivor group had significantly lower APACHE II and SOFA than the non-survivor group, 15.6±3.3 vs 23.4±3.3, p<0.001, and 8.4±2.1 VS 12.5±2.2, p <0.001, respectively. Regarding baseline nutritional status, most of the survivors had SGA class A while the non-survivors had SGA class B and C. Enteral nutrition was the preferred route of nutrition administration in both groups and only 3% of patients were kept nil per os (NPO) at the time of CRRT initiation (Table 1). All patients received either standard formula enteral nutrition (standard blenderized diet) by tube feeding or standard parenteral nutrition (Oliclinomel^**®**^ or SmofKabiven^**®**^).

**Table 1:** Baseline characteristics between survivor and non-survivor group.

Demographic	Survivor (n = 27)	Non-survivor (n = 43)	P value
Age (years)	57.3±16.8	62.8±16.8	0.18
Gender, male (%)	20 (74.1)	27 (62.8)	0.32
Primary treating service –no. (%)			0.16
Medical	13 (48.1)	28 (65.)	
Surgical	14 (51.9)	15 (34.9)	
Comorbid disease –no. (%)	23 (85.2)	41 (95.3)	0.13
Diabetes mellitus	11 (40.7)	11 (25.5)	0.18
Hypertension	11 (40.7)	24 (55.8)	0.22
Coronary artery disease	7 (25.9)	8 (18.6)	0.46
Valvular heart disease	2 (7.4)	2 (4.6)	0.62
Stroke	0 (0.0)	9 (20.9)	0.01
Liver disease	7 (25.9)	12 (27.9)	0.85
Malignancy	6 (22.2)	11 (25.6)	0.75
HBV infection	1 (3.7)	3 (6.9)	0.56
HCV infection	1 (3.7)	1 (2.3)	0.73
HIV infection	0 (0.0)	1 (2.3)	0.42
BMI (kg/m^2^)	21.2±2.3	20.3±4.5	0.32
Baseline serum creatinine(mg/dl)	1.12±0.33	1.14±0.38	0.79
Serum creatinine when initiating dialysis (mg/dl)	4.07±1.61	3.26±1.19	0.01
APACHE II	15.6±3.3	23.4±3.3	<0.001
SOFA	8.4±2.1	12.5±2.2	<0.001
SGA –no. (%)			0.002
Class A	17 (62.9)	12 (27.9)	
Class B	10 (37.1)	19 (44.2)	
Class C	0 (0.0)	12 (27.9)	
Nutrition route			0.11
Enteral (EN)	23 (85.2)	25 (58.1)	
Parenteral (PN)	2 (7.4)	10 (23.2)	
EN + PN	2 (7.4)	6 (13.9)	
NPO	0 (0.0)	2 (4.8)	
Cause of AKI –no. (%)			0.04
Ischemic	16 (59.2)	12 (27.9)	
Nephrotoxic	0 (0.0)	1 (2.3)	
Sepsis	11 (40.8)	27 (62.8)	
Multifactorial	0 (0.0)	3 (7.0)	
Indication of dialysis –no. (%)			0.51
Metabolic acidosis	6 (22.2)	11 (25.5)	
Hyperkalemia	2 (7.4)	1 (2.3)	
Volume overload	5 (18.5)	12 (27.9)	
Oliguria, anuria	12 (44.4)	12 (27.9)	
Uremia	0 (0.0)	1 (2.3)	
Hypercatabolic state	2 (7.5)	6 (14.1)	
Vasopressor –no. (%)	24 (88.8)	42 (97.6)	0.12
Dobutamine	5 (18.5)	15 (34.9)	0.14
Dopamine	14 (51.8)	32 (74.4)	0.05
Epinephrine	2 (7.4)	10 (23.2)	0.08
Norepinephrine	20 (74)	39 (90.7)	0.06

HBV: hepatitis B virus, HCV: hepatitis C virus, HIV: human immunodeficiency virus, BMI: body mass index, APACHE II: Acute Physiology and Chronic Health Evaluation II, SOFA: Sequential Organ Failure Assessment, SGA: subjective global assessment, NPO: nil per os, AKI: acute kidney injury.

### Macronutrients and Clinical Outcomes

**Fig 2:**
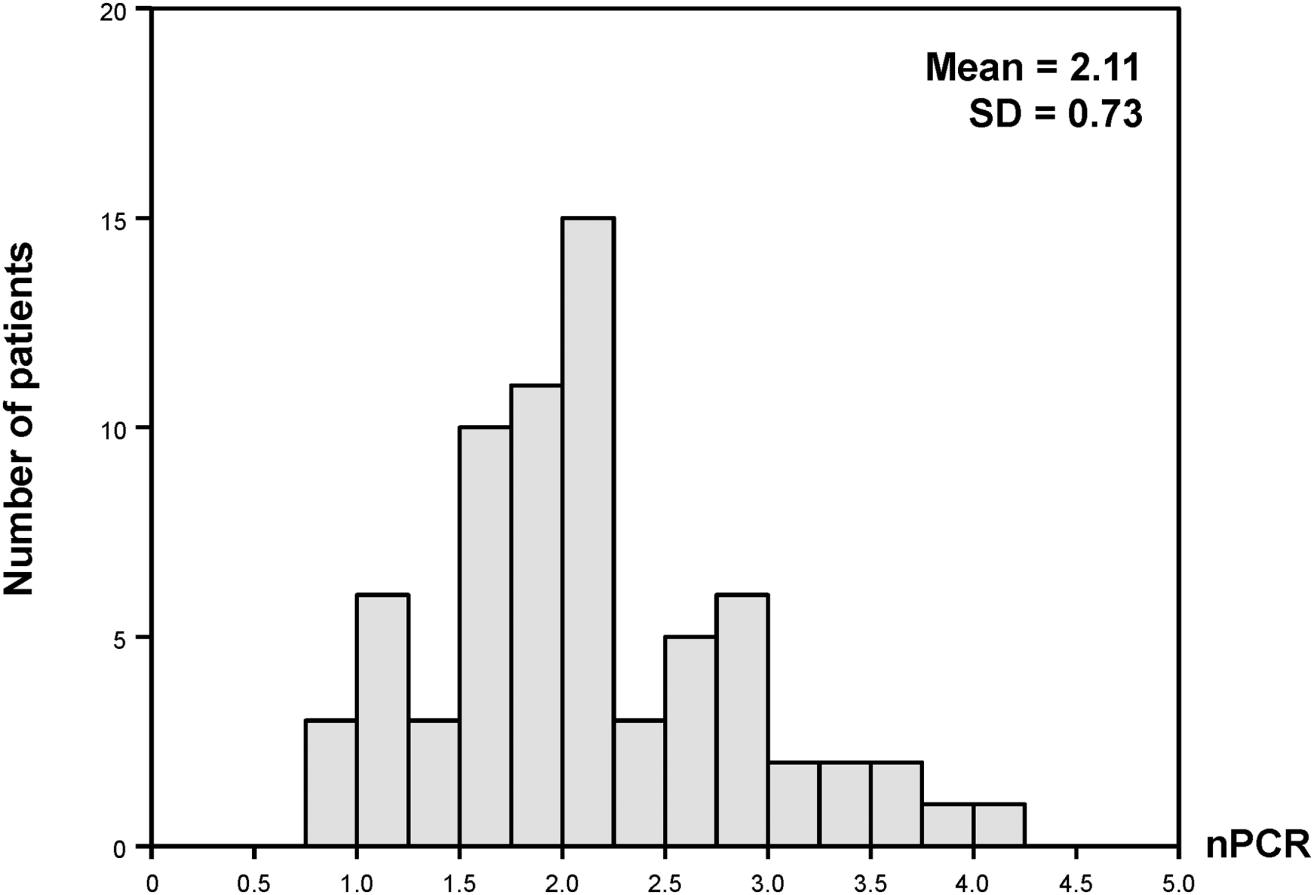
Distribution of normalized protein catabolic rate (nPCR).

Overall, the alteration of DPI during the first three days of CRRT were 0.5±0.3, 0.6±0.3, and 0.7±0.4 g/kg/day, respectively. The mean changes of nPCR were 2.2±0.7 (day 1), 2.1±0.7 (day 2), 2.1±0.8(day 3), g/kg/day. Fig 2 shows the distribution of nPCR, most of nPCR ranged from 1 to 3 g/kg/day.CRRT patients had negative nitrogen balance (-10.8, -10.3, and -11.5 g/day by day 1, 2, and 3, respectively). Serum albumin was quite low (mean serum albumin 3.0 g/dL) while CRP level was high (mean CRP = 115.6 mg/L).

When comparing macronutrient parameters between the survivor and the non-survivor groups, only DPI and serum albumin in the survivor group were significantly higher than the non-survivor group, 0.8±0.2 vs 0.5±0.3 g/kg/day p<0.001, and 3.2±0.5 vs 2.9±0.5 g/dL, p = 0.03, respectively (Table 2).

**Table 2:** Comparison of macronutrient/micronutrient parameters between survivor and non-survivor group.

**Macronutrients**	**Survivor (n = 27)**	**Non-survivor (n = 43)**	**P value**
DPI (g/kg/day)	0.8 (0.2)	0.5 (0.3)	<0.001
Nitrogen balance (g/day)	-9.3 (6.1)	-11.8 (6.8)	0.13
nPCR(g/kg/day)	2.1 (0.6)	2.0 (0.8)	0.76
Serum albumin (g/dL)	3.2 (0.5)	2.9 (0.5)	0.03
CRP (mg/L)	119.4 (49.5)	112.8 (42.3)	0.72
**Micronutrients**	**Survivors (n = 13)**	**Non-survivors (n = 24)**	**P value**
Zinc (mg/L)	0.86 (1.27)	1.06 (1.66)	0.71
Selenium (mg/L)	0.09 (0.05)	0.08 (0.07)	0.80
Copper (mg/L)	0.61 (0.54)	0.80 (0.65)	0.39

Values are given as mean (standard deviation). nPCR: normalized protein catabolic rate, CRP: C-reactive protein.

Regarding renal outcome, the patients who had renal recovery had significantly higher level of serum albumin than renal non-recovery, 3.3±0.5 vs 2.9±0.4 g/kg/day, p = 0.01. The levels of other macronutrients were comparable between both groups (Table 3).

**Table 3:** Comparison of macronutrient parameters between dialysis independent group and dialysis dependent group.

Macronutrients	Dialysis independent group (n = 15)	Dialysis dependent group (n = 12)	P value
nPCR(g/kg/day)	2.1±0.6	2.2±0.6	0.72
Protein intake (g/day)	41.8±10.9	46.7±15.7	0.34
Protein intake per body weight (g/kg/day)	0.7±0.2	0.8±0.3	0.35
Nitrogen balance (g/day)	-8.7±7.1	-10.1±4.6	0.57
Serum albumin (g/dL)	3.3±0.5	2.9±0.4	0.01
CRP (mg/L)	114.8±73.7	123.3±22.6	0.79

Values are given as mean (standard deviation). nPCR: normalized protein catabolic rate, CRP: C-reactive protein.

In the model of nutrition markers for predicting mortality, DPI showed the highest AUC score at 0.69. Combining DPI, nitrogen balance, nPCR, serum albumin, and CRP can increase the AUC score to 0.78 (Table 4).

**Table 4:** Clinical model of nutritional parameters for predicting mortality.

Model	Nutritional markers	AUC (95% CI)
A	DPI per body weight	0.69 (0.55, 0.84)
B	Nitrogen balance	0.60 (0.45, 0.75)
C	nPCR	0.51 (0.36, 0.66)
D	Albumin	0.54 (0.32, 0.77)
E	CRP	0.50 (0.27, 0.73)
A+B+C+D+E		0.78 (0.60, 0.96)

nPCR: normalized protein catabolic rate,CRP: C-reactive protein.

Table 5 shows the univariable and multivariable analyses to predict mortality. When adjusted for differences in serum albumin, severity score (APACHE II and SOFA), and serum creatinine at the time of CRRT initiation, the DPI independently predicted mortality (odds ratio = 4.62, 95% CI 1.48–14.47, p = 0.009).

**Table 5:** Analysis of nutritional makers to predict mortality.

Nutritional markers	Odds ratio (95% CI) unadjusted	P value	Odds ratio (95% CI) adjusted	P value
DPI (per 0.2 g/kg/day)	1.91 (1.26,2.88)	0.002	4.62 (1.48,14.47)	0.009
Nitrogen balance (per 1 g/day)	1.06 (0.98,1.15)	0.13	0.92 (0.78,1.08)	0.30
nPCR (per 1 g/kg/day)	1.11 (0.57,2.15)	0.76	3.48 (0.78,15.52)	0.10
Albumin (per 1 g/dL)	3.23 (1.12,9.31)	0.03		
CRP (per 1 mg/L)	1.00 (0.99,1.02)	0.71	6.20 (0,∞)	0.98

Adjusted by albumin, APACHE II, SOFA, serum creatinine at CRRT initiation. nPCR: normalized protein catabolic rate, CRP: C-reactive protein.

### Micronutrients and Clinical Outcomes

The mean baseline patient’s serum zinc (0.89 mg/L, normal 0.7–1.6 mg/L), selenium (0.07 mg/L, normal 0.07–0.13 mg/L), and copper (0.74 mg/L, normal 0.70–1.6 mg/L) during the first 3 days of CRRT were in normal range and remained on the following days, except for serum copper in the third day of CRRT (S1 Table). There were no significant differences of baseline micronutrients between the survivor and non-survivor groups (Table 2). Regarding the dialysis status among survivors, there were no difference of micronutrient levels between the dialysis dependent and independent groups. There were no correlations between micronutrients and ICU length of stay, hospital length of stay, and duration of CRRT (data not shown).

## Discussion

The present prospective observational study in severe AKI requiring CRRT has demonstrated that macronutrients especially low DPI and low serum albumin were associated with the mortality. The patients in both groups had high nPCR values. Micronutrients were unchanged and did not influence the mortality.

From the clinical model of nutritional markers to predict mortality, DPI had the highest AUC-ROC among the nutritional markers (Table 4). Furthermore, the low DPI was still the only nutritional markers that showed association with mortality in the model adjusted for disease severity score (Table 5). Since the patients in our study are quite sick (mean APACHE II was about 20) at the time of CRRT initiation, nutritional intake was restricted in some patients during the critical period. Inadequate DPI in highly catabolic AKI patients may cause progressive lean body mass loss leading to protein-energy wasting (PEW), cachexia, and poor clinical outcome [14]. Of note, the mean DPI in our study was 0.6 g/kg/day which was quite similar to a recent *post hoc* analysis of the RENAL study [13]. In contrast to our results, such study found that a lower DPI was not associated with increased 90-day mortality or any secondary outcomes (28-day mortality, death in the ICU, in-hospital death, RRT outcome, duration of ICU and hospital stays, duration of mechanical ventilation and RRT, dialysis status at day 90, and any new organ failures). Indeed, the RENAL study was designed to test the effect of intensity of CRRT and patient outcomes, but not aimed to study the association of nutrition parameters and clinical outcome. Moreover, in the RENAL study, there was no collection of effluent volume to directly measure protein breakdown during CRRT treatment as performed in the present study.

Interestingly, our study found that the DPI and serum albumin of survivors were significantly higher than non-survivors. These findings correspond with guidelines for the provisional and assessment of nutritional support therapy in the adult critically Ill patients bySociety of Critical Care Medicine (SCCM) and American Society for Parenteral and Enteral Nutrition (A.S.P.E.N.) [15].

In the present study, we also found that hypoalbuminemia was related to the higher mortality rate and higher dialysis dependence (Tables 2 and 3). Hypoalbuminemia is associated with malnutrition and systemic inflammation since albumin is one of the negative acute-phase protein[16].Data from animal studies revealed that albumin can bind with platelet-activating factor and nitrogen oxide, turning to S-nitroso-albumin which has renal vasodilatory effect and help maintaining the renal perfusion [17]. Moreover, albumin can also indirectly protect renal cellular damage by binding with reactive oxygen species, and can activate the DNA synthesis via multiple signaling pathways [18].

In our study, the serum levels of micronutrients were unaltered. This finding is similar to some previous studies, which reported a very small amount of trace elements loss from CRRT [19,20]. However, Berger et al. reported the significant losses and negative balances of selenium, copper, and thiamine from CRRT, which contributed to low plasma concentrations [21]. At present, there is no optimal dosing recommendation for trace element supplementation in AKI patients from the international guidelines.

Of interest, the nPCR in CRRT patients in the present study (Table 6) was higher than the values in previous studies [10–13] and the 2012 Kidney Disease Improving Global Outcome (KDIGO) AKI guideline recommendation (2.1 vs 1.7 g/kg/day)[22]. There are possible explanations why nPCR is high during CRRT. First, most of the previous studies came from the postoperative surgical patients treated by CAVH which had low efficacy in uremic toxin removal [23–27]. Second, the dialysis technique related factor, the current CRRT technique used high flux biocompatible synthetic membranes which can contribute to the significant higher protein losses. Third, the patient related factor, most of the patients in our cohort had multiple organ dysfunction, more protein wasting, and might be sicker (mean APACHE II score was 23 in non-survivor group) than the patients in the previous studies.

**Table 6:** Summary previous nutrition studies in CRRT patients including current study.

Study	CRRT modality	N	Macronutrient parameters		Patient outcomes
			DPI (g/kg/d)	nPCR(g/kg/d)	
Chima CS,1993 [10]	CAVH	19	1.4	1.7	N/A
Macias WL, 1996 [11]	CVVH	40	N/A	1.4	N/A
Leblanc M, 1998 [12]	CVVH, CVVHD,CVVHDF	38	N/A	1.75	N/A
Bellomo R, 2014 [13]	CVVHDF	1,508	0.5	N/A	Yes
Our study, 2015	CVVH	70	0.6	2.1	Yes

CAVH: continuous arteriovenous hemofiltration, CVVH: continuous venovenous hemofiltration, CVVHD: continuous venovenous hemodialysis, CVVHDF: continuous venovenous hemodiafiltration, DPI: dietary protein intake, nPCR: normalized protein catabolic rate, N/A: not available

Our study has several strengths. First, we collected the effluent fluid for calculating direct nPCR in all cases. Moreover, we could demonstrate that nPCR in the patients undergoing CRRT was significantly higher than the KDIGO 2012 guideline recommendation [22]. Second, regarding to the instability of CRRT patients, our study measured dynamic change macronutrients/ micronutrients parameters in three times point to get the most reliable data for analysis. Third, this is the first report of all macronutrients/ micronutrients parameters with the clinical outcome (Table 6).

Admittedly, our study has some limitations. First, this is an observational study which could not control all of the confounding factors. However, we have tried to adjust possible confounding factors in the analysis predictor model (Table 5). Second, since our study is not the randomized controlled study, we cannot clearly demonstrate whether DPI is the cause of poor clinical outcome or just the marker of severity of illness. We still need the large randomized controlled study. However, this study is the first step for conducting further validation studies. Third, for the micronutrients analysis, we did not measure the trace elements in the effluent fluid. We cannot show the transmembrane clearance of trace elements by the CRRT as demonstrated in some previous studies [19,20,28]. Lastly, we have tested nutritional markers at early periods (on the first three days of CRRT initiation)and could not answer the predictive value of nutrition markers at other time points. Nevertheless, risk stratification and prognostication using nutrition markers is likely to be useful only when nutritional markers are measured early.

## Conclusions

Critically ill patients requiring CRRT have very high catabolism according to the high nPCR. DPI is a good predictor of patient survival while serum albumin is a good prognosticator of renal outcome. A large randomized controlled trial is needed to prove this concept.

## Supporting Information

S1 Table Micronutrient levels on the first three days of CRRT. (DOCX)
