# Supplementary material for: The Impact of Macro-and Micronutrients on Predicting Outcomes of Critically Ill Patients Requiring Continuous Renal Replacement Therapy
Source: PLoS One. 2016 Jun 28;11(6):e0156634. doi: 10.1371/journal.pone.0156634 (PMC4924859; doi:10.1371/journal.pone.0156634)
Supplement: S1 Table — (DOCX) [file pone.0156634.s001.docx]

**Supplement**

**S1 Table. Micronutrient levels on the first three days of CRRT**

| **Micronutrients** | **Serum concentration** | | | **Reference range** |
| --- | --- | --- | --- | --- |
|  | **Day 1**  **(n = 37)** | **Day 2**  **(n = 25)** | **Day 3**  **(n = 15)** |  |
| Zinc (mg/L) | 0.99 (1.52) | 0.93 (1.30) | 0.75 (1.43) | 0.70-1.60 |
| Selenium (mg/L) | 0.08 (0.06) | 0.07 (0.06) | 0.07 (0.06) | 0.07-0.13 |
| Copper (mg/L) | 0.73 (0.61) | 0.81 (0.55) | 0.67 (0.65) | 0.70-1.60 |

Values are given as mean (standard deviation).
